# The Brief Lexington Attachment to Pets Scale: measurement invariance in India, Italy, Poland, and Russia

**DOI:** 10.1186/s40359-025-03080-6

**Published:** 2025-07-09

**Authors:** Sofya K. Nartova-Bochaver, Sofia I. Reznichenko, Paweł Larionow, Silvia Ariccio, Jakub Jankowski, Shanmukh V. Kamble, Oriana Mosca, Monika Oziemblewska, Appasaheb C. Patil, Ekaterina K. Scherba, Michalina Sołtys, Timothy P. Johnson

**Affiliations:** 1 Department of Psychology, National Research University Higher School of Economics (HSE University), Myasnitskaja Str., 20, 101000 Moscow, Russia; 2 Faculty of Psychology, Kazimierz Wielki University, Leopolda Staffa Str. 1, 85–867, Bydgoszcz, Poland; 3 University College of Professional Education in Wroclaw, Plac Powstańców Śląskich 1 Str, 53‑329, Wrocław, Poland; 4 Utrecht University, Heidelberglaan 6, Utrecht, Netherlands; 5 Department of Psychology, Karnatak University, Karnataka State, Dharwad, Pavate Nagar, 580003 India; 6 Department of Pedagogy, Psychology, Philosophy, University of Cagliari, Via Università, 40, Sardinia, Cagliari CA 09124 Italy; 7 Department of Public Policy, Management, and Analytics, University of Illinois at Chicago, 412 S. Peoria St., 115 CUPPAH (MC 350), Chicago, IL 60607 USA

**Keywords:** Pet attachment, Lexington Attachment to Pets Scale, Measurement invariance, Culture, Psychometric properties

## Abstract

Pet attachment is a phenomenon widely studied in various cultures, but little attention has been devoted to whether it varies across cultures. This topic requires a valid invariant instrument to measure pet attachment. Thus, the aim of this study was cross-cultural examination of the Lexington Attachment to Pets Scale (LAPS). A total of 906 respondents from India, Italy, Poland, and Russia took part in the study. The initial version of LAPS has 23 items and three factors: General attachment, People substitution, and Animal rights/welfare. We expected that (1) LAPS retains its structure across the four countries studied, and that (2) the level of pet attachment varies from country to country. The confirmatory factor analysis did not support the original three-factor structure in all the countries, with specific deviations from the initial structure in each participating country. After many iterations, we decided to investigate a new optimal structure of LAPS. The final one-factor model consisted of eleven items was supported, and this modification was labelled the Brief Lexington Attachment to Pets Scale (B-LAPS). B-LAPS had high omega internal consistency (0.84–0.88), and has demonstrated configural, full metric, and partial scalar measurement invariance across countries. It was shown that the highest score in pet attachment was found in Poland, followed by India, Italy, and Russia. Future research is needed to further validate the external validity of B-LAPS and assess its usefulness in research, animal-assisted therapy, and education.

**Supplementary Information:**

The online version contains supplementary material available at 10.1186/s40359-025-03080-6.

## Background

Pets as companions have long been a part of the life of modern people. Pet ownership has increased significantly during the past three decades globally, with more than half of people internationally having at least one pet [4]. The passion for pets implies the existence of attachment to them although ownership and attachment are not completely synonymous. Pet attachment (PA) includes affective ties that pet owners feel; it is an emotional bond to pets, manifesting in care of them, willingness to spend money and other resources on them, and missing them in the case of separation from them or death [34, 61, 88].

Although PA is considered in different ways [6, 17, 28, 34, 38, 63, 72, 89], the Johnson et al. [34] conceptualization seems to be most systemic and convincing. Within the framework of this approach, PA is characterized by the level of positive affect regarding pets (general attachment), the pets’ ability to replace people (people substitution), and the willingness of people to provide pets with social rights (animal rights/animal welfare). In addition, PA is understood in regard to which biological species the pet belongs [20, 56]; thus, PA seems to be a multidimensional phenomenon, for many reasons, attracting researchers’ attention. First, animal-assisted therapy, which is highly effective in solving psychological problems of a wide range, has become very popular [3, 8, 14, 40]. Secondly, animal rights movements are increasing all over the world, which also needs careful investigation into factors associated with animal harm [15, 81–83]. Thirdly, due to current demographic changes, animals are increasingly seen as a substitute for loved ones or family members, including children [80].

Over the years, research has accumulated evidence that PA can have beneficial effects on pet owners (“pet effect”) in various areas of human positive functioning, such as emotional state, communication, improving somatic health, etc. [2, 10, 12, 23, 26, 33, 35, 40, 42, 48, 52–54, 58, 59, 67–69, 71, 75, 84]. Recent studies, however, have begun to highlight the “dark sides” of having pets, like unhealthy attachment [5] or pathological pet hoarding [56], in which cases companion animals can develop behaviors undesirable to the owner (aggression, separation problems), provoking social tension between pet owners and non-owners [58, 77]. Some more trends, contradictory in terms of social and personal well-being, are replacing people with pets [61, 75] and pet parenting (the investment of money, emotion, and time in companionship with animals) [29, 78–80]. So, in some cases, PA may aggravate problematic mental health [39, 76]. In sum, nowadays, it has become widely accepted that PA does not unequivocally yield benefits, and as a consequence, it seems imperative that PA should be investigated by means of recently developed or re-validated tools.

## Pet attachment in the cultural context

Attitudes toward pet ownership are known to vary by geographic, economic, cultural, and demographic variables [4, 32, 65, 79]. Whereas gender, age, race, and other demographic predictors form inconsistent patterns with PA [6, 44, 47, 66, 70, 71, 74], there is evidence that pet culture may depend on the prevailing cultural values in the country as a whole [18, 24]. Although pet owning and being attached to pets are not synonymous, in the typical case pets do not appear in the absence of attachment. Countries differ regarding the percentage of pet ownership and preferred types of pets: the USA and Latin American countries tend to have higher rates of pet ownership [16]. Furthermore, the objects of attachment differ, and pets popular in one culture may not arouse interest and desire to have them at home in another. Dogs, followed by cats, are the most popular pets in Latin America and Poland; in Russia, France, and the USA, people most often have cats [55]. Pet fish are most popular in China, and pet birds in Turkey. Some religions also matter for attitude toward animals, placing a strong emphasis on the non-harm of animals [57]. We believe that PA is the foundation of pet culture. To promote cross-cultural research on PA, it is necessary to ensure that the tools used in research allow for the comparison of attachment in different countries, i.e. have cultural invariance. To our knowledge, none of their existing tools have passed such a test so far.

## Previous adaptations of the Lexington Attachment to Pets Scale

The Lexington Attachment to Pets Scale (LAPS) is one of the most popular measurement instruments for the study of PA [34]. Despite having been developed for work with cat and dog owners, the tool is also used to study attachment as a general attitude [87], with specific animals (for example, dolphins) [85], or with animals living in a zoo [30].

LAPS is a 23-item self-reported questionnaire. It consists of three subscales: eleven-item General attachment (e.g., “I play with my pet quite often”), seven-item People substitution (e.g., “I love my pet because it never judges me”), and five-item Animal rights/welfare (e.g., “Pets deserve as much respect as humans do”).

Developed in the USA [34], LAPS is widely used in many countries: Brazil [1, 43, 46], China [42], Croatia [41], Germany [25, 36], India [80], Italy [64, 73], Mexico [62], Pakistan [69], Russia [51], and South Africa [13]. In Croatia, Germany, and Italy, the original three-factor structure of LAPS was kept. In some cases, researchers modified the scale; in others, they extracted some single statements from LAPS to use them in a new questionnaire [45].

Shams et al. [69] extended the four-point Likert scale to the five-point one. Esterhuyzen [13] obtained a two-factor structure. Wilshaw [87] modified the scale by adding seven negative statements. As part of our previous experience with LAPS in a Russian sample, Nartova-Bochaver et al. [51] shortened the scale to eight items (LAPS-Short Form), and identified a four-factor structure, with the following subscales: Closeness to pets, Pets’ rights, Empathy from pets, and Happiness from pets. Thus, for all its attractiveness to researchers, LAPS is still not stable in all applications. Perhaps this is caused by the content of the statements themselves, which do not seem different enough to really form a multifactorial structure. For instance, some items that in the original version belong to different subscales are semantically close paraphrases: “I believe my pet is my best friend” from the General attachment subscale, and “I consider my pet to be a friend” from the People substituting subscale. Furthermore, some statements appear to be semantically related to several scales. For example, the item “I feel that my pet is a part of my family”, which in the original version was included in the Animal rights subscale, in our opinion, more corresponded to the General attachment subscale, as it emphasizes the closeness of the relationship with the pet, rather than the desire to equalize the rights of humans and animals. The inconsistency of the structure of the original version of the scale can also be seen in the high cross-correlations of some items with all subscales: for example, the item “My pet understands me” strongly loaded (λ > 0.3) on three scales simultaneously [34, p. 168]. Finally, most items have a pronounced “ceiling effect”, i.e., they are highly skewed, which may lead to structural inconsistency, especially in the case of multigroup factor analysis.

Moreover, strict verification of the scale suggests other shortcomings. Some researchers have noted conceptual and psychometric limitations of LAPS [64, 87, 88]. Hence, LAPS focuses on the affective facet of the bond only, whereas other relevant aspects of the relationship are not considered. In addition, the relationship with specific pet species cannot be measured by LAPS, demonstrating non-universality of this tool. Finally, psychometric examination of LAPS has not been systematic: in most cases, researchers limited their work to the translation and examination of the reliability of LAPS only, without evaluation of its validity [41, 46, 62, 64, 73].

Given these criticisms of LAPS, many researchers have chosen to develop new tools (e.g., 43, 64, etc.]. Following our previous useful results on LAPS obtained in the Russian sample [51], in our opinion, however, a more constructive option would be the revalidation of the questionnaire using an international sample. This would make it possible (1) to compare previously obtained results, (2) to revise the concept of PA according to its contemporary manifestations (3) to prepare a psychometrically sound research instrument, and (4) to compare the PA levels in different countries.

## The current study

This study examines the structural validity of LAPS in India, Italy, Poland, and Russia. These countries have different levels of wealth and represent different cultures [74]. Research shows that individualism/collectivism, among all of Hofstede’s dimensions of culture [27], has the most significant links with general attitudes toward nature [50]. According to the Inglehart-Welzel values framework [21], India leans toward traditionalism and survival, Italy toward secularism and self-expression, Poland toward traditionalism and self-expression, and Russia toward secularism and survival. Hence, if we succeed in developing an invariant scale structure in these very different countries, it may both result in a reliable standardized measure and strengthen the validity of the PA construct.

## Methods

### Participants and procedure

A total of 906 respondents from India, Italy, Poland, and Russia took part in the study. The data were collected in 2022 and 2023. The Russian part of the current international data were used in a previous study [51]. Participation was voluntary; the respondents provided demographic information: age, sex, level of education, country of residence, and type of pets. The samples were not completely equivalent and balanced in terms of demographic characteristics (see Table 1). In Russia, the participants were granted an academic credit.

**Table 1:** Description of the samples

Country	N	Age	Gender	Culture	GDP in 2022	Data collection and language
India	138	M_age_ = 19.57, SD_age_ = 2.81	46.4% men	Collectivistic (Ind = 48) T Su	2.39	pencil-and-paper, in person, in English
Italy	153	M_age_ = 26.61, SD_age_ = 7.05	58.8% men	Individualistic (Ind = 76) S Se	34.16	online via Prolific, in Italian
Poland	306	M_age_ = 33.01, SD_age_ = 10.34	8.5% men	Individualistic (Ind = 60) T Se	18.23	online (predominantly) and paper-and-pencil, via Google Forms, in Polish
Russia	236	M_age_ = 26.41, SD_age_ = 9.84	24.6% men	Collectivistic (Ind = 39) S Su	15.35	online, via 1 ka.si service, in Russian

*Ind* Individualism index by Hofstede, *GDP* Gross Domestic Product per capita (current USD), *S/T* Secularism/Traditionalism according to Inglehart-Welzel values, *Su/Se* Survival/Self-expression according to Inglehart-Welzel values

The criteria for inclusion in the sample were: age 18–65 years old, permanent residence in one of the listed countries, and owning one or more pets. Participants who did not meet the inclusion criteria or who owned any type of pet other than dogs or cats were excluded from the sample. After removing outliers from each subsample (4.5%; based on the Mahalanobis distance criterion at *p* < 0.001) and participants who did not meet the inclusion criteria (8.1%), the aggregate sample size was 833, with 138 participants in India, with 153 in Italy, with 306 in Poland, and 236 in Russia (see Table 1). According to the N:q rule of thumb for both exploratory and confirmatory factor analyses, which requires 5–10 cases for each estimated parameter, the Russian and Polish sample sizes are sufficient, while the Italian and Indian sample sizes are somewhat smaller than required [37]. However, a model-free a priori RMSEA-based power analysis [49] showed that a sample size of *N* = 104 was sufficient to detect misspecifications of a structure model involving 227 degrees of freedom (which corresponds to the original three-factor LAPS model with 23 manifest variables) at RMSEA effect size of 0.05 on alpha = 0.05 with a power of 80%. Our study's minimum sample of 138 individuals per country provides a power of 0.97 to reject “close fit” (i.e., RMSEA H0 ≤ 0.05) when in the real population there is not-close fit (i.e., RMSEA H1 ≥ 0.08).

### Measures

Initially, we used LAPS in its full original form, including 23 items and three subscales: General attachment, People substitution, and Animal rights/welfare [34]. The statements are scored on a four-point scale ranging from 0 (*strongly disagree*) to 3 (*strongly agree*), with higher scores indicating higher levels of PA.

### Translation of LAPS

With permission from the developers of LAPS, it was translated into Russian and Polish by native speakers according to ISPOR requirements [86]. After all the statements were translated back, they were discussed and edited until a consensus was reached. In Italy, we used the translation by Riggio et al. [64]. As their version referred to dogs and not to pets, for our study, we replaced the word *cane* (dog) or *cani* (dogs) with pet (*animale domestico*) or pets (*animali domestici*). In India, the survey was completed in English.

### Analytic strategy

Statistical analysis was carried out using MS Excel and R (v. 4.2.3) packages semPower (v. 2.1.1) for a-priori power analyses, MVN 5.9 for assessing multivariate normality, MBESS 4.9.3 for calculating effect sizes and confidence intervals, psych 2.3.9 for conducting exploratory factor analyses and reliability analysis as well as providing descriptive statistics, lavaan 0.6–16 and semTools 0.5–6 for conducting confirmatory factor analysis and measurement invariance tests, and stats (v. 3.6.2) for performing analysis of variance. The analysis was carried out in the following sequence. First, we planned to check the individual test items regarding their distributions and inter-total correlations. Then, the original structure of LAPS in individual countries was be examined. As we were unable to keep the original structure, we searched for a new optimal structure. Finally, the measurement invariance of the original (or modified) questionnaire was be tested across countries.

## Results

### Testing statistical assumptions and item analysis

The main sample (*N* = 833) had no missing values. Most items showed a slight bias towards higher scores and had a ceiling effect. From this point of view, the most problematic in all samples were items 14, 19–22. A floor effect was detected for items 6 and 9 in the Russian sample, and for item 2 in the Polish one. The maximum possible scores of items were observed in all samples. In Italy, no minimum scores were found for items 19, 20, and 22 and in India for 14–16, 18–20, and 22–23. Multivariate (Mardia’s test) and univariate normality (Anderson–Darling test) were not met in any sample, *p* < 0.001. Distributions were mostly negatively asymmetric and leptokurtic. In terms of the absolute values, the range of skewness and Pearson’s kurtosis were acceptable to prove normal univariate distribution [19] and did not exceed ± 2, except for the Polish sample, in which items 14, 16, 18–23 had extremely high skewness and kurtosis values.

Under the Classical Test Theory framework, we also analyzed item difficulty and discrimination indices, and item-total correlations. The majority of items had acceptable difficulty (15–85%) in the Russian and Italian samples, but not in the Indian and Polish ones. Discrimination for most items was acceptable (> 0.2) [36]. The average inter-total correlations were 0.56, 0.62, 0.23, and 0.59 for India, Italy, Poland, and Russia, respectively (see Additional file 1).

### Testing the original structure of LAPS in individual countries

We performed confirmatory factor analysis (CFA) with the robust maximum likelihood rescaling-based estimator to analyze the original factor structure of LAPS. A set of commonly used goodness-of-fit indicators was used to interpret the results: CFI, TLI, RMSEA, and SRMR. CFI and TLI values exceeding 0.95 indicate a good model fit [31]. An RMSEA value not greater than 0.06 suggests a good fit, while SRMR values smaller than 0.08 indicate an acceptable fit.

In all countries, the original three-factor correlated model had unacceptable fit indices (Additional file 2, Model 1), due to high error covariances between items 7–11, 10–12–22, 6–9–20, 19–22, and 3–14 or cross-correlations of these items with other subscales. After removing items 3, 6, 7, 12, 22, which contribute the most noise, the model fit to the data improved significantly in all samples (Additional file 2, Model 2). In addition, several more error covariances between items were found individually in each sample, especially in Poland and Russia. However, the removal of these items led to a decrease in fit indices in other cultures, so this modification was not approved.

The modified factor structure is as close as possible to the original LAPS structure and fits well with data from India and Italy but has poor performance with the Polish and Russian data. It is important to note that the Heywood case was observed in the Indian structural model (the covariance matrix was not positive definite), which may artificially inflate the fit indices. Model 2 has other limitations as well. First, in all samples, the correlations between the three subscales were too high (India 0.82–0.99; Italy 0.86–0.93; Russia 0.87–0.90, and Poland 0.68–0.87; average correlation 0.86), which presumably indicates in favor of a unidimensional structure. Secondly, some items still have poor skewness and kurtosis, which reduces the accuracy of the model. Thirdly, some items in Indian and Russian cultures have low loadings on their subscales (> 0.30), reducing their internal reliability. This deep analysis forced us to return to exploratory research to determine the optimal number of subscales in the available data.

### Searching for a new optimal structure of LAPS

The parallel analysis based on principal axis factor analysis and the Velicer MAP criterion were used to determine the optimal number of factors [11]. Exploratory factor analysis using Pearson correlations, MLR estimator, and oblique rotation was conducted to assign the items to the extracted factor. Since the Indian and Italian samples were too small to divide them into separate subsamples for EFA and CFA, we had to perform both types of analysis on the same data; the Russian and Polish samples were randomly divided in half: 50% each for the EFA and CFA. The results of the Kaiser–Meyer–Olkin test for each sample ranged from 0.87 to 0.92 and had significant Bartlett's Test of Sphericity statistics (*p* < 0.001), indicating that the construction of scales of different variables is suitable for further analyzing the relationships between variables.

The parallel analysis revealed one factor in the Indian sample (first three eigenvalues: 8.31, 0.72, 0.66), two factors in the Italian sample (eigenvalues: 8.76, 0.92, 0.57), and three factors in the Polish (eigenvalues: 0.97, 1.55, 0.78) and Russian samples (eigenvalues: 7.60, 0.87, 0.77). Only in the Polish sample was the eigenvalue of the second factor greater than one (the Kaiser criterion), indicating that the extraction of multiple factors does not significantly increase the proportion of variance explained. According to the MAP criterion, the Indian and Russian factor structures were unidimensional, whereas the optimal number of factors for the Italian and Polish samples was two.

EFA results indicate that the two-factor model does not optimally describe the data from all four countries: in different cultures, the same items load on different factors; in certain cultures, some items have low (< 0.3) factor loadings on both factors or load on two factors at once; the cumulative explained variance did not exceed 0.45 in any case (see Additional file 3). Similar trends are true for the three-factor model, in which the third factor included only two items (7 and 11) related to the presentation of a pet to others. Taking into account the instability of the two-factor and three-factor models in different cultures, and the problems identified during the analysis of the original factor structure, it was decided to focus on the unidimensional LAPS model.

According to CFA results, the unidimensional model, consisting of 23 LAPS items, had a poor fit to the data (Additional file 2, Model 3). Items 7 and 9 had the lowest factor loadings across all cultures and were removed first. Then, based on modification indices, the items with multiple, high error covariances with other items, and significant deviations from the normal distribution were sequentially excluded: 2, 3, 4, 5, 6, 12, 16, 20–22. As expected, these changes led to significant improvements in fit across all cultures (Additional file 2, Model 4). The reversed item 8 (“I think my pet is just a pet”) in the Indian sample had a relatively low factor loading (0.29), however, its removal significantly worsened fit indices in other cultures. Therefore, it was decided to keep it. All variances in the model were positive and statistically significant. Thus, the final one-factor model consisted of eleven items. Due to this strong shortening of the scale, the new modification was called the Brief Lexington Attachment to Pets Scale (B-LAPS; see Additional file 4).

The internal consistency of B-LAPS was assessed with McDonald’s omega (ω) on the full samples (i.e. both CFA and EFA subsamples in Russia and Poland) and was satisfactory (> 0.7) in all countries [22]. Descriptive statistics of B-LAPS in the countries studied are shown in Table 2. Despite the removal of items with deviations from normality, kurtosis values were still high in the Polish sample, and the small sample size in both India and Italy led to a narrowing of the range of values.

**Table 2:** Descriptive statistics of B-LAPS across countries

	N	Min	Max	M (SD)	SE	Me	Asymmetry	Kurtosis	McDonalds Omega [95% CI]
India	138	0,55	3	2.46 (0.44)	0.04	2.55	−1.25	1.67	0.84 [0.78–0.88]
Italy	153	0.55	3	2.24 (0.50)	0.04	2.36	−0.77	0.18	0.88 [0.84–0.91]
Poland	306	0	3	2.54 (0.49)	0.03	2.68	−2.43	4.55	0.87 [0.83–0.92]
Russia	236	0	3	2.09 (0.60)	0.04	2.18	−0.86	0.58	0.86 [0.83–0.89]

*SE* standard errors, *Me* Median; McDonald’s omega’s confidence intervals have been estimated through bootstrapping with 1,000 replicates

### Measurement invariance testing across countries

Testing the invariance of B-LAPS across countries was carried out via Multigroup Confirmatory Factor Analysis (MGCFA). MGCFA contained three assessments of equivalence with increasing constraints: configural (CFA model was fitted for each group separately, without any equality constraints, to test whether the same factorial structure holds across all groups), metric (factor loadings were assumed to be equal across groups), and scalar (both the factor loadings and intercepts were assumed to be equal across groups). Evaluation of the invariance was conducted by the assessment of changes in fit indices: ΔCFI and ΔTLI less than 0.01, ΔRMSEA less than 0.015, and ΔSRMR less than 0.03 [9].

Table 3 shows that configural invariance was confirmed which assumed that the overall factor structure is identical across countries. The model comparison test (configural vs. metric) suggested full metric invariance (ΔCFI = − 0.006, ΔTLI = − 0.002, ΔRMSEA = 0.000, ΔSRMR = 0.026), indicating that factor loadings were comparable in all countries. However, scalar invariance was not achieved, because all differences in CFI and TLI values significantly exceeded their thresholds: ΔCFI = − 0.040, ΔTLI = − 0.032, ΔRMSEA = 0.017, ΔSRMR = 0.013. Based on modification indices, the intercepts of items 11, 19, and 23 were freed for testing partial scalar invariance. The intercept partially invariant model showed a non-significant difference in fit: ΔCFI = − 0.018, ΔTLI = 0.013, ΔRMSEA = 0.008, ΔSRMR = 0.006. Since partial scalar invariance was achieved, it allowed the comparison of scale means across countries.

**Table 3:** Measurement invariance of B-LAPS across countries (*N* = 833)

Model	*χ*^*2*^/df	CFI	TLI	RMSEA (90% CI)	SRMR
1. Configural invariance	252.27/176***	0.971	0.964	0.046 (0.034; 0.056)	0.041
2. Metric invariance	298.46/206***	0.965	0.962	0.046 (0.036; 0.056)	0.067
Δ 2–1	46.19/30	−0.006	−0.002	0.000	0.026
3. Scalar invariance	431.63/236***	0.925	0.930	0.063 (0.055; 0.071)	0.080
Δ 3–2	133.17/30	−0.040	−0.032	0.017	0.013
4. Partial scalar invariance ^a^	364.75/227***	0.957	0.959	0.054 (0.045–0.063)	0.073
Δ 4–2	66.29/21	−0.008	−0.003	0.008	0.006

^a^ freed intercepts of items 11, 19, and 23

The magnitude of the latent mean structure difference of B-LAPS was specified using Cohen’s *d*, measuring the effect size of differences in means, where *d* > 0.2 is considered as a small effect, *d* = 0.5 is medium, and *d* = 0.8 ≤ a significant effect (Cohen, 1988). Since the Russian sample had a smaller factor mean, we fixed its latent mean at zero and standard deviation at one whereas the latent means and standard deviations of other groups were freely estimated. The latent means, compared by a one-way ANOVA, significantly differed across countries, F_(3, 829)_ = 37.05, *p* < 0.001. Post-hoc comparisons using Tukey’s test showed that Russian respondents had the lowest PA score (*M* = 2.09, *SD* = 0.60), and Polish respondents had the highest one (*M* = 2.54, *SD* = 0.49, *p* > 0.001; Cohen’s *d* = 0.84). Italian respondents (*M* = 2.24, *SD* = 0.50) were more strongly attached to their pets than Russian respondents (*p* = 0.026; Cohen’s *d* = 0.27), but less than Indian (*M* = 2.46, *SD* = 0.44, *p* = 0.002, Cohen’s *d* = 0.47) and Polish respondents (*p* < 0.001; Cohen’s *d* = 0.61). The means of respondents from India and Poland were not significantly different (*p* = 0.422; Cohen’s *d* = 0.17).

## Discussion

### Examination of the original structure of LAPS

This study was aimed to examine the psychometric properties of LAPS in four countries varying in economic development and cultures, namely, in India, Italy, Russia, and Poland. Despite LAPS being widely used, to our knowledge, this is the first systematic examination of its psychometric properties and cross-cultural invariance. The original scale consisted of 23 items and had a three-factor structure, with three subscales: General attachment, People substitution, and Animal rights/welfare. In contrast to expectations, the original three-factor structure was not supported in any country. Possible explanations include that firstly, LAPS was developed in 1992, and since then, the psychometric standards have been radically changed and become more demanding [22, 36]. Despite internal consistency and factor structure being described and item response theory modeling presented in the initial LAPS version, confirmatory factor analysis was not completed at that time. In addition, the lack of observed cross-cultural universality of LAPS may also be caused by the sample differences in their demographic characteristics.

Secondly, in the initial version, some items duplicated each other's meaning and seemed to have high social desirability. So, in contemporary society, pets are considered a significant social value, and we can see a ceiling effect in most items indicating a positive shift in responses: even if people do not like pets, they may not admit it so as not to annoy the community. Thirdly, the very content of the PA has developed new features over the past thirty years: owning pets has become more prestigious, and the owners are spending more resources on caring for them now [52, 60, 80]. Finally, the initial version of LAPS was developed in USA, a country strongly differing from the countries participating in the current research: it is more prosperous and, over the years, has demonstrated a very high level of pet ownership whereas in some other countries, only relatively recently have people begun to have funds for pet care, and to promote humane treatment of animals [16]. So, it could also damage the ecological validity of LAPS when using it in countries other than the USA.

### The modified version of LAPS (B-LAPS)

In this regard, we revised the factor structure of LAPS and reduced the number of items by half, eliminating statements that were psychometrically weak and variable across different cultures. The new unidimensional version is called the Brief Lexington Attachment to Pets Scale (B-LAPS) and consists of 11 items reflecting different aspects of PA. While analyzing the attribution of the items to the factor, most of the excluded items belonged to the factor People substitution (6 out of 12). This may indicate that contemporary pet owners do not assume that communicating with pets is equivalent to communicating with people. As for the items kept in the modified version, most of them were from the General attachment (7 out of 11) subscale, 3 from the Animal rights/welfare subscale, and one from the People substitution subscale. These results are in line with those obtained by Templer et. al. [72] who emphasized love as the core of PA, and Lago et al. [38] who identified companionship. Based on this, we can assume that the nucleus of PA is a positive emotional attitude, and not the social role or function of a pet, which seems to vary over time and depend somewhat on culture. It is not surprising that the core content reflects those components and fits into the framework of a healthy attachment.

### Measurement invariance of B-LAPS and score comparisons across countries

B-LAPS has a good fit to the data from all four cultures and high internal reliability. The configural, metric, and partial measurement invariance of the tool across countries is supported, allowing the comparison of PA levels in various cultures. The countries studied differ significantly in their level of PA, with the highest mean values observed in Poland, followed by India, then Italy, and the lowest in Russia. Surprisingly, this result contrasts with the data from a GfK poll [16] indicating that Russians have the highest percentage of cat owners, and that, in the Asia region, there is the least number of pet owners. At the same time, to have a pet is not the same as to love the pet, and B-LAPS measures just PA.

At first glance, it seems that PA level is on average higher in the more prosperous countries with individualistic and self-realization cultures (Italy and Poland), but the number of countries (i.e., four) in our cross-cultural sample was too small for evidence-based generalizations. At the same time, in India the PA score was high. Therefore, the comparison results should be treated as tentative. It is possible that not a single factor, like values, religion, or income per capita, determines the level of PA but the interaction of these variables or some less obvious factors may determine these outcomes. We can speculate that demographic policy practiced in some Asian countries, for instance, India, with a limit on the number of children, encourages people to have a pet just in order to replace other objects of care [86]. In turn, in the countries without such limitations, like Italy and Russia, the level of PA observed is lower, which may be affected by general motives to have a pet. Despite many controversial issues, we can conclude that a modified version of LAPS can be used for further research in the countries participating in this study.

## Limitations and directions for future research

Our research has additional limitations, which define the next steps of research in this area. In the current study, we were limited only to the examination of B-LAPS structural validity and measurement invariance, without its convergent and divergent validity. These lines for future research might then be a follow-up to the current study. The systematic validation of B-LAPS should be replicated before connections between PA and various indicators of person’s positive functioning and well-being, specifying the role of pets in the contemporary world, are examined. Furthermore, our sample was heterogeneous and not balanced by gender, age, or preferred pet, which did not allow us to verify the measurement invariance of B-LAPS with respect to sex, age and type of pet. In addition, in India and Italy, due to not very large samples, both EFA and CFA were conducted on the same sample, and the factor loadings found in the Indian sample were relatively low. These shortcomings may be addressed by extending the sample in future research, opening up the prospects for a detailed study of the connection between PA and the cultural dimensions of countries.

## Conclusions

In this cross-cultural study, the original three-factor structure of the 23-item LAPS was not supported. Therefore, we developed a modified, shorter version of LAPS comprised of eleven items, which was invariant across the four countries. This provides new research perspectives and allows researchers to return to earlier results for their re-analysis and reinterpretation. We believe that B-LAPS, once its construct and predictive validity are rigorously established, can be used by researchers, practitioners, and educators for studying and optimizing pet-person interactions.

## Supplementary Information

Additional file 1. Descriptive statistics of the Lexington Attachment to Pets Scale items. The Table

Additional file 2. Goodness-of-fit indices for the original and modified LAPS models in different cultures. The Table

Additional file 3. Factor loadings of the Lexington Attachment to Pets Scale items in different countries for a two-factor model. The Table

Additional file 4. The Brief Lexington Attachment to Pets Scale: Extended English, Corresponding Reduced English, Italian, Polish, and Russian Versions. The Table

## Data Availability

The data that support the findings of this study are available from the second corresponding author (S. N.-B.) upon reasonable request.

## References

[1] Albuquerque NS, Costa DB, dos Reis RG, Sessegolo NS, Moret-Tatay C, Irigaray TQ. Adaptation and psychometric properties of Lexington Attachment to Pets Scale: Brazilian version (LAPS-B). J Vet Behav. 2023;61:50–6.

[2] Allen K. Are pets a healthy pleasure? The influence of pets on blood pressure. Curr Dir Psychol Sci. 2003;12:236–9.

[3] Altschiller D. Animal-assisted therapy. Bloomsbury Publishing USA; 2011.

[4] American Pet Products Association. Data and Research [Internet]. American Pet Products Association; 2023 [cited 2024 Nov 24]. Available from: https://www.americanpetproducts.org/

[5] Andrews SL. The inventory of pet attachment: Development and validation [dissertation]. Texas A&M University; 1992.

[6] Bagley DK, Gonsman VL. Pet attachment and personality type. Anthrozoös. 2005;18:28–42.

[7] Bosacki S, Tardif-Williams CY, Roma RPS. Children’s and adolescents’ pet attachment, empathy, and compassionate responding to self and others. Adolescents. 2022;2:493–507.

[8] Chandler CK. Animal-assisted therapy in counseling: Third edition. Taylor and Francis; 2017.

[9] Chen FF. Sensitivity of goodness of fit indexes to lack of measurement invariance. Struct Equ Modeling. 2007;14:464–504.

[10] Chen NR, Majeed NM, Hartanto AY, et al. Human–animal interaction and human prosociality: A meta-analytic review of experimental and correlational studies. Anthrozoös. 2024;37:269–88.

[11] Cohen J. Statistical power analysis for the behavioral sciences. Hillside, NJ: Lawrence Earlbaum Associates; 1988.

[12] Douglas VJ, Kwan MY, Gordon KH. Pet attachment and the interpersonal theory of suicide. Crisis. 2021;44:14–20. 34463529 10.1027/0227-5910/a000822

[13] Esterhuyzen M. The psychometric properties of the Coleman Dog Attitude Scale and Owner-Pet Relationship Scale-Modified: A South African study [dissertation]. Stellenbosch University; 2020.

[14] Fine AH. Handbook on animal-assisted therapy: Foundations and guidelines for animal-assisted interventions. Elsevier; 2019.

[15] Freeman CP. The human animal earthling identity: Shared values unifying human rights, animal rights, and environmental movements. University of Georgia Press; 2020.

[16] GfK. Pet ownership: Global GfK survey [Internet]. 2016 [cited 2024 Nov 24]. Available from: https://cdn2.hubspot.net/hubfs/2405078/cms-pdfs/fileadmin/user_upload/country_one_pager/nl/documents/global-gfk-survey_pet-ownership_2016.pdf

[17] Gosling SD, Sandy CJ, Potter J. Personalities of self-identified “dog people” and “cat people.” Anthrozoös. 2010;23:213–22.

[18] Gray PB, Young SM. Human-pet dynamics in cross-cultural perspective. Anthrozoös. 2011;24:17–30.

[19] Gravetter F, Wallnau L. Essentials of statistics for the behavioral sciences. 8th ed. Belmont, CA: Wadsworth; 2014.

[20] Haddon C, Burman OH, Wilkinson A. Love in Cold Blood: Are Reptile Owners Emotionally Attached to Their Pets? Anthrozoös. 2021;34:739–49.

[21] Haerpfer C, Inglehart R, Puranen B. World Values Survey Wave 7 (2017–2024) Cross-National Data-Set [Internet]. 2023 [cited 2024 Nov 24]. Available from: 10.14281/18241.18

[22] Hair J, Black W, Anderson R. Multivariate Data Analysis: A Global Perspective. Pearson; 2010.

[23] Hawkins RD, Robinson C, Brodie ZP. Child-Dog Attachment, Emotion Regulation and Psychopathology: The Mediating Role of Positive and Negative Behaviours. Beh Sci. 2022;12(4):109. 10.3390/bs12040109 PMC9027944 35447681

[24] Herzog HA. Biology, Culture, and the Origins of Pet-Keeping. Anim Behav Cogn. 2014;1(3):296–308.

[25] Hielscher B, Gansloßer U, Froboese I. Attachment to Dogs and Cats in Germany: Translation of the Lexington Attachment to Pets Scale (LAPS) and Description of the Pet Owning Population in Germany. Hum Anim Interact Bull. 2019;3:1–18.

[26] Hoffman CL, Stutz K, Vasilopoulos T. An examination of adult women’s sleep quality and sleep routines in relation to pet ownership and bedsharing. Anthrozoös. 2018;31:711–25.

[27] Hofstede Insights. Hofstede Insights Country Comparison Tool [Internet]. 2023 [cited 2024 Nov 24]. Available from: https://www.hofstede-insights.com/n.a

[28] Holcomb R, Williams CR, Richards SP. The elements of attachment: Relationship maintenance and intimacy. J Delta Soc. 1985;2:28–34.

[29] Horecka K, Neal S. Critical problems for research in animal sheltering, a conceptual analysis. Front Vet Sci. 2022;9: 804154. 35433910 10.3389/fvets.2022.804154 PMC9010978

[30] Hosey G, Birke L, Melfi V. Measuring the strength of human–animal bonds in zoos. Anthrozoös. 2018;31:273–81.

[31] Hu LT, Bentler PM. Cutoff criteria for fit indexes in covariance structure analysis: Conventional criteria versus new alternatives. Struct Equ Modeling. 1999;6:1–55.

[32] Ivanski C, Lo RF, Mar RA. Pets and politics: Do liberals and conservatives differ in their preferences for cats versus dogs? Collabra Psychol. 2021;7(1):28391.

[33] Janssens M, Eshuis J, Jacobs N. The pet-effect in daily life: An experience sampling study on emotional wellbeing in pet owners. Anthrozoös. 2020;33:579–88.

[34] Johnson TP, Garrity TF, Stallones L. Psychometric evaluation of the Lexington Attachment to Pets Scale (LAPS). Anthrozoös. 1992;5:160–75.

[35] Kanat-Maymon Y, Wolfson S, Roth G. The benefits of giving as well as receiving need support in human–pet relations. J Happiness Stud. 2021;22:1441–57.

[36] Kartik A, Neeraj R. Itemized analysis of questions of multiple choice question (MCQ) exam. Int J Sci Res. 2013;2:279–80.

[37] Kyriazos T. Applied psychometrics: Sample size and sample power considerations in factor analysis (EFA, CFA) and SEM in General. Psychology. 2018;9:2207–30.

[38] Lago D, Kafer R, Connell C. Assessment of favorable attitudes toward pets: Development and preliminary validation of self-report pet relationship scales. Anthrozoös. 1988;1:240–54.

[39] Lass-Hennemann J, Schäfer SK, Michael T. The relationship between attachment to pets and mental health: The shared link via attachment to humans. BMC Psychiatry. 2022;22(1):586. 36057711 10.1186/s12888-022-04199-1 PMC9441033

[40] le Roux MC, Wright S. The relationship between pet attachment, life satisfaction, and perceived stress: Results from a South African online survey. Anthrozoös. 2020;33:371–85.

[41] Levačić J. Pokušaj validacije Adaptirane Lexington skale privrženosti kućnim ljubimcima. Suvrem Psihol. 2009;12(2):391–405.

[42] Lu J, Ren E, Zhang N. The role of pet attachment in alleviating the negative effects of loneliness on a health-promoting lifestyle: An empirical study based on threshold effects for pet owners. Int J Older People Nurs. 2023;18(5): e12554. 37461157 10.1111/opn.12554

[43] Luchesi SH, Machado DS, Otta E. Psychometric validation of the Brazilian version of the Pet Attachment Questionnaire (PAQ): An examination of predictors of attachment styles among cat owners. Appl Anim Behav Sci. 2022;256: 105769.

[44] Maharaj Y. Is the humanisation trend wagging the SA pet care market? [Internet]. Insight Survey; 2017 [cited 2024 Nov 24]. Available from: http://www.insightsurvey.co.za/blog/humanisation-trend-wagging-sa-pet-care-market

[45] Marsa-Sambola F, Muldoon J, Currie C. The Short Attachment to Pets Scale (SAPS) for Children and Young People: Development, psychometric qualities and demographic and health associations. Child Indic Res. 2016;9:111–31.

[46] Martins MF. Grau de apego dos proprietários com os animais de companhia segundo a Escala Lexington Attachment to Pets. BJVRAS. 2014;50(5):364–9.

[47] Marx MB, Stallones LB, Johnson TP. Demographics of pet ownership among US adults 21 to 64 years of age. Anthrozoös. 1988;2(1):33–7.

[48] McConnell AR, Brown CM, Martin CE. Friends with benefits: On the positive consequences of pet ownership. J Pers Soc Psychol. 2011;101:1239–52. 21728449 10.1037/a0024506

[49] Moshagen M, Bader M. semPower: General power analysis for structural equation models. Behav Res Methods. 2024;56(4):2901–22. 37950114 10.3758/s13428-023-02254-7 PMC11522193

[50] Nartova-Bochaver SK, Donat M, Clayton S. The role of environmental identity and individualism/collectivism in predicting climate change denial: Evidence from nine countries. J Environ Psychol. 2022;84: 101899.

[51] Nartova-Bochaver SK, Larionow P, Scherba EK. Attachment to pet and love for people—is there a connection? Sotsial’naya psikhologiya i obshchestvo = Social Psychology and Society. 2024;15(1):156–70.

[52] Ngai N. Homemade pet celebrities: The everyday experience of micro-celebrity in promoting the self and others. Celebrity Stud. 2023;14:437–54.

[53] Ogata N, Weng HY, Messam LL. Temporal patterns of owner-pet relationship, stress, and loneliness during the COVID-19 pandemic, and the effect of pet ownership on mental health: A longitudinal survey. PLoS ONE. 2023;18. 10.1371/journal.pone.0284101 PMC10132673 37099517

[54] Oliva JL, Johnston KL. Development of the Pet Owner Connectedness Scale (POCS). Anthrozoös. 2022;35:545–57.

[55] OnePoll. Case studies [Internet]. OnePoll; 2023 [cited 2024 Nov 24]. Available from: https://www.onepoll.us/portfolio/justfoodfordogs-pet-love/

[56] Patronek G. Animal hoarding: A third dimension of animal abuse. In: Ascione FR, editor. The International Handbook of Animal Abuse and Cruelty: Theory, Research, and Application. Purdue University Press; 2008. p. 221–40.

[57] Perry SL, Burge RP. How religion predicts pet ownership in the United States. J Sci Stud Relig. 2020;59:190–201.

[58] Podberscek AL. Positive and negative aspects of our relationship with companion animals. Vet Res Commun. 2006;30(Suppl 1):21–7.

[59] Poresky RH, Hendrix C. Differential effects of pet presence and pet-bonding on young children. Psychol Rep. 1990;67:51–4. 2236419 10.2466/pr0.1990.67.1.51

[60] Prato-Previde E, Basso Ricci E, Colombo ES. The complexity of the human–animal bond: Empathy, attachment and anthropomorphism in human–animal relationships and animal hoarding. Animals. 2022;12(20):2835. 36290219 10.3390/ani12202835 PMC9597799

[61] Rajaram SS, Garrity TF, Marx MB. Bereavement—Loss of a pet and loss of a human. Anthrozoös. 1993;6:8–16.

[62] Ramírez MTG, Berumen LdCQ, Hernández RL. Psychometric properties of the Lexington Attachment to Pets Scale: Mexican version (LAPS-M). Anthrozoös. 2014;27:351–9.

[63] Rauktis ME, Hoy-Gerlach J, Bickel L. Preliminary findings of a ten-item scale to assess the commitment of low-income owners to their companion animals. Anthrozoös. 2021;34:109–26.

[64] Riggio G, Piotti P, Mariti C. The dog–owner relationship: Refinement and validation of the Italian C/DORS for dog owners and correlation with LAPS. Animals. 2021;11(8):2166. 34438624 10.3390/ani11082166 PMC8388506

[65] Scanlon L, Hobson-West P, Stavisky J. Homeless people and their dogs: Exploring the nature and impact of the human–companion animal bond. Anthrozoös. 2021;34:77–92.

[66] Schoenfeld-Tacher R, Kogan LR, Wright ML. Comparison of strength of the human-animal bond between Hispanic and non-Hispanic owners of pet dogs and cats. J Am Vet Med Assoc. 2010;236:529–34. 20187816 10.2460/javma.236.5.529

[67] Schulz C, König HH, Hajek A. Differences in self-esteem between cat owners, dog owners, and individuals without pets. Front Vet Sci. 2020;7:55. 32984412 10.3389/fvets.2020.00552 PMC7492270

[68] Schwarzmueller-Erber G, Maier M, Kundi M. Pet attachment and wellbeing of older-aged recreational horseback riders. Int J Environ Res Public Health. 2020;17(6):1865. 32183083 10.3390/ijerph17061865 PMC7143422

[69] Shams H, Ishrat F, Kiyani MM. Age-based relationships among loneliness, pet attachment support, wellbeing, and quality of life in pet owners: A socio-emotional and neurological rehabilitation perspective. Biomed J Sci Tech Res. 2021;34(4):27035–9.

[70] Spicer S. Pets and their people: A sociological investigation into the pet-keeping practices of two demographically diverse samples of South Africans [dissertation]. University of the Western Cape; 2012.

[71] Stallones L, Johnson TP, Marx MB. Quality of attachment to companion animals among US adults 21 to 64 years of age. Anthrozoös. 1990;3(3):171–6.

[72] Templer DI, Salter CA, Veleber DM. The construction of a Pet Attitude Scale. Psychol Rec. 1981;31:343–8.

[73] Testoni I, De Cataldo L, Zamperini A. Pet loss and representations of death, attachment, depression, and euthanasia. Anthrozoös. 2017;30:135–48.

[74] The World Bank. The World Bank Group [Internet]. 2023 [cited 2024 Nov 24]. Available from: https://data.worldbank.org/indicator/NY.GDP.PCAP.CD01269.x

[75] Triebenbacher SL. The companion animal within the family system: The manner in which animals enhance life within the home. In: Handbook on Animal-Assisted Therapy. Elsevier Inc.; 2000. p. 357–74.

[76] Trigg J, Thompson K, Smith B, Bennett P. Exploring risk propensity through pet-attachment diversity in natural hazard contexts. Hum Anim Interact Bull. 2016;4(1):54–81.

[77] Volkan K. Hoarding and animal hoarding: Psychodynamic and transitional aspects. Psychodyn Psychiatry. 2021;49:24–47. 33635102 10.1521/pdps.2021.49.1.24

[78] Volsche S. Pet parenting in the United States: Investigating an evolutionary puzzle. Evol Psychol. 2021;19(3):14747049211038296. 34569356 10.1177/14747049211038297 PMC10355291

[79] Volsche S, Frailey F, Nittono H. Sex and parental status impacts human-to-pet attachment and caregiving attitudes and behaviors in a Japanese sample. Anthrozoös. 2023;36:309–21.

[80] Volsche S, Mukherjee R, Rangaswamy M. The difference is in the details: Attachment and cross-species parenting in the United States and India. Anthrozoös. 2022;35:393–408.

[81] Wauthier LM, Williams JM. Understanding and conceptualizing childhood animal harm: A meta-narrative systematic review. Anthrozoös. 2022;35:165–202.

[82] Wauthier LM, Farnfield S, Williams JM. The role of attachment in children’s relationships with pets: From pet care to animal harm. Hum Anim Interact. 2022;1.

[83] Wauthier L, Williams JM. A qualitative study of children’s accounts of cruelty to animals: Uncovering the roles of trauma, exposure to violence, and attachment. J Interpers Violence. 2022;37:NP6405–NP43. 10.1177/0886260520928640 PMC9092908 32597294

[84] Wells DL. The state of research on human–animal relations: Implications for human health. Anthrozoös. 2019;32:169–81.

[85] Welsh T, Ward S. Visitor attachment to dolphins during an interaction programme: Are there implications to dolphin behavior? Zoo Biol. 2021;40:551–62. 34254346 10.1002/zoo.21634

[86] Wild D, Grove A, Martin M, Eremenco S, McElroy S, Verjee-Lorenz A, Erikson P. Principles of good practice for the translation and cultural adaptation process for patient-reported outcomes (PRO) measures: report of the ISPOR task force for translation and cultural adaptation. V. in Health. 2005;8;2: 94–104. 10.1111/j.1524-4733.2005.04054.x 15804318

[87] Wilshaw J. Measuring attachments between dogs and their owners [dissertation]. University of Exeter; 2010.

[88] Zaparanick TL. A confirmatory factor analysis of the Lexington Attachment to Pets Scale [dissertation]. Knoxville: University of Tennessee; 2008.

[89] Zilcha-Mano S, Mikulincer M, Shaver PR. An attachment perspective on human-pet relationships: Conceptualization and assessment of pet attachment orientations. J Res Pers. 2011;45:345–57.

