# Supplementary material for: The Brief Lexington Attachment to Pets Scale: measurement invariance in India, Italy, Poland, and Russia
Source: BMC Psychol. 2025 Jul 9;13:754. doi: 10.1186/s40359-025-03080-6 (PMC12239344; doi:10.1186/s40359-025-03080-6)
Supplement: Supplementary file 1 — Additional file 1. Descriptive statistics of the Lexington Attachment to Pets Scale items. The Table [file 40359_2025_3080_MOESM1_ESM.docx]

**Additional file 1.**

**Descriptive statistics of the Lexington Attachment to Pets Scale items**

| Item | Mean | SD | Med | Min | Max | 25th | 75th | Sk | Ku | Diff | Disc | ITC | L/H scores, % |
| --- | --- | --- | --- | --- | --- | --- | --- | --- | --- | --- | --- | --- | --- |
| *India (N=138)* | | | | | | | | | | | | | |
| LAPS1 | 1.98 | 0.80 | 2 | 0 | 3 | 2 | 3 | -0.49 | -0.23 | 0.66 | 0.36 | 0.63 | 4.7 / 27.1 |
| LAPS2 | 1.98 | 0.87 | 2 | 0 | 3 | 2 | 3 | -0.59 | -0.31 | 0.66 | 0.33 | 0.53 | 7.0 / 30.2 |
| LAPS3 | 2.56 | 0.63 | 3 | 0 | 3 | 2 | 3 | -1.33 | 1.38 | 0.85 | 0.25 | 0.52 | 0.08 / 63.6 |
| LAPS4 | 2.62 | 0.54 | 3 | 0 | 3 | 2 | 3 | -1.37 | 2.50 | 0.87 | 0.24 | 0.63 | 0.08 / 65.1 |
| LAPS5 | 2.16 | 0.87 | 2 | 0 | 3 | 2 | 3 | -0.73 | -0.35 | 0.72 | 0.37 | 0.59 | 4.7 / 42.6 |
| LAPS6 | 2.63 | 0.61 | 3 | 0 | 3 | 2 | 3 | -1.64 | 2.43 | 0.87 | 0.20 | 0.50 | 0.08 / 70.0 |
| LAPS7 | 2.50 | 0.75 | 3 | 0 | 3 | 2 | 3 | -1.54 | 1.99 | 0.83 | 0.17 | 0.35 | 3.1 / 62.8 |
| LAPS8 | 2.34 | 0.86 | 3 | 0 | 3 | 2 | 3 | -1.07 | 0.13 | 0.78 | 0.27 | 0.43 | 3.9 / 55.8 |
| LAPS9 | 2.37 | 0.88 | 3 | 0 | 3 | 2 | 3 | -1.25 | 0.59 | 0.79 | 0.18 | 0.26 | 5.4 / 58.9 |
| LAPS10 | 2.37 | 0.70 | 2 | 0 | 3 | 2 | 3 | -1.07 | 1.15 | 0.79 | 0.31 | 0.56 | 2.3 / 48.8 |
| LAPS11 | 2.39 | 0.66 | 2 | 0 | 3 | 2 | 3 | -0.95 | 1.01 | 0.79 | 0.23 | 0.53 | 1.6 / 48.1 |
| LAPS12 | 2.48 | 0.62 | 3 | 0 | 3 | 2 | 3 | -1.15 | 1.94 | 0.82 | 0.26 | 0.53 | 1.6 / 53.5 |
| LAPS13 | 2.37 | 0.69 | 2 | 0 | 3 | 2 | 3 | -0.93 | 0.61 | 0.79 | 0.33 | 0.63 | 1.6 / 48.8 |
| LAPS14 | 2.69 | 0.52 | 3 | 1 | 3 | 2 | 3 | -1.47 | 1.23 | 0.89 | 0.28 | 0.70 | 0.0 / 72.9 |
| LAPS15 | 2.68 | 0.53 | 3 | 1 | 3 | 2 | 3 | -1.38 | 0.93 | 0.89 | 0.25 | 0.61 | 0.0 / 71.3 |
| LAPS16 | 2.52 | 0.56 | 3 | 1 | 3 | 2 | 3 | -0.63 | -0.68 | 0.84 | 0.31 | 0.70 | 0.0 / 55.8 |
| LAPS17 | 2.45 | 0.68 | 3 | 0 | 3 | 2 | 3 | -1.29 | 1.94 | 0.81 | 0.20 | 0.42 | 2.3 / 54.3 |
| LAPS18 | 2.64 | 0.57 | 3 | 1 | 3 | 2 | 3 | -1.32 | 0.74 | 0.88 | 0.30 | 0.72 | 0.0 / 69.0 |
| LAPS19 | 2.80 | 0.39 | 3 | 2 | 3 | 3 | 3 | -1.53 | 0.34 | 0.93 | 0.19 | 0.69 | 0.0 / 80.6 |
| LAPS20 | 2.77 | 0.48 | 3 | 1 | 3 | 3 | 3 | -2.06 | 3.50 | 0.92 | 0.24 | 0.70 | 0.0 / 80.6 |
| LAPS21 | 2.24 | 0.98 | 3 | 0 | 3 | 2 | 3 | -0.97 | -0.31 | 0.74 | 0.33 | 0.41 | 7.8 / 55.0 |
| LAPS22 | 2.65 | 0.56 | 3 | 1 | 3 | 2 | 3 | -1.40 | 0.98 | 0.88 | 0.27 | 0.62 | 0.0 / 70.5 |
| LAPS23 | 2.79 | 0.46 | 3 | 1 | 3 | 3 | 3 | -2.09 | 3.66 | 0.93 | 0.19 | 0.61 | 0.0 / 81.4 |
| *Italy (N=153)* | | | | | | | | | | | | | |
| LAPS1 | 1.75 | 0.92 | 2 | 0 | 3 | 1 | 2 | -0.19 | -0.89 | 0.58 | 0.54 | 0.76 | 9.2 / 24.3 |
| LAPS2 | 1.78 | 0.91 | 2 | 0 | 3 | 1 | 2 | -0.44 | -0.57 | 0.59 | 0.43 | 0.68 | 11.1 / 21.7 |
| LAPS3 | 1.59 | 0.60 | 2 | 0 | 2 | 1 | 2 | -1.16 | 0.30 | 0.53 | 0.27 | 0.59 | 5.9 / 0.0 |
| LAPS4 | 1.55 | 0.95 | 2 | 0 | 3 | 1 | 2 | -0.01 | -0.95 | 0.51 | 0.51 | 0.73 | 14.5 / 18.4 |
| LAPS5 | 1.43 | 0.87 | 1 | 0 | 3 | 1 | 2 | 0.05 | -0.71 | 0.47 | 0.28 | 0.52 | 14.5 / 11.2 |
| LAPS6 | 1.82 | 0.91 | 2 | 0 | 3 | 1 | 3 | -0.26 | -0.86 | 0.60 | 0.43 | 0.64 | 7.9 / 27.0 |
| LAPS7 | 2.20 | 0.77 | 2 | 0 | 3 | 2 | 3 | -0.53 | -0.64 | 0.73 | 0.22 | 0.46 | 1.3 / 40.8 |
| LAPS8 | 2.03 | 0.82 | 2 | 0 | 3 | 1 | 3 | -0.41 | -0.63 | 0.67 | 0.35 | 0.58 | 3.3 / 32.2 |
| LAPS9 | 1.71 | 0.59 | 2 | 0 | 2 | 2 | 2 | -1.88 | 2.31 | 0.57 | 0.15 | 0.40 | 7.2 / 0.0 |
| LAPS10 | 1.98 | 0.85 | 2 | 0 | 3 | 1 | 3 | -0.46 | -0.52 | 0.66 | 0.37 | 0.63 | 5.3 / 30.3 |
| LAPS11 | 2.13 | 0.81 | 2 | 0 | 3 | 2 | 3 | -0.45 | -0.81 | 0.71 | 0.31 | 0.53 | 2.0 / 38.8 |
| LAPS12 | 1.86 | 0.80 | 2 | 0 | 3 | 1 | 2 | -0.37 | -0.30 | 0.62 | 0.38 | 0.69 | 5.3 / 21.1 |
| LAPS13 | 2.19 | 0.80 | 2 | 0 | 3 | 2 | 3 | -0.89 | 0.44 | 0.73 | 0.40 | 0.69 | 4.6 / 39.5 |
| LAPS14 | 2.62 | 0.58 | 3 | 0 | 3 | 2 | 3 | -1.47 | 2.12 | 0.87 | 0.28 | 0.58 | 0.07 / 67.1 |
| LAPS15 | 2.26 | 0.69 | 2 | 0 | 3 | 2 | 3 | -0.76 | 0.59 | 0.75 | 0.36 | 0.78 | 2.0 / 39.5 |
| LAPS16 | 2.44 | 0.67 | 3 | 0 | 3 | 2 | 3 | -0.92 | 0.15 | 0.81 | 0.35 | 0.76 | 0.07 / 53.9 |
| LAPS17 | 2.23 | 0.63 | 2 | 0 | 3 | 2 | 3 | -0.38 | 0.02 | 0.74 | 0.21 | 0.54 | 0.6 / 33.6 |
| LAPS18 | 2.50 | 0.60 | 3 | 0 | 3 | 2 | 3 | -0.96 | 0.75 | 0.83 | 0.32 | 0.71 | 0.7 / 55.3 |
| LAPS19 | 2.67 | 0.53 | 3 | 1 | 3 | 2 | 3 | -1.34 | 0.82 | 0.89 | 0.25 | 0.67 | 0.0 / 70.4 |
| LAPS20 | 2.68 | 0.49 | 3 | 1 | 3 | 2 | 3 | -1.10 | -0.03 | 0.89 | 0.22 | 0.58 | 0.0 / 69.7 |
| LAPS21 | 2.61 | 0.68 | 3 | 0 | 3 | 2 | 3 | -1.84 | 3.12 | 0.87 | 0.19 | 0.48 | 2.0 / 70.4 |
| LAPS22 | 2.56 | 0.55 | 3 | 1 | 3 | 2 | 3 | -0.82 | -0.38 | 0.85 | 0.26 | 0.63 | 0.0 / 60.0 |
| LAPS23 | 2.31 | 0.75 | 2 | 0 | 3 | 2 | 3 | -0.77 | -0.27 | 0.77 | 0.37 | 0.71 | 1.3 / 48.0 |
| *Poland (N=306)* | | | | | | | | | | | | | |
| LAPS1 | 2.35 | 0.75 | 3 | 0 | 3 | 2 | 3 | -0.92 | 0.15 | 0.78 | 0.61 | 0.35 | 1.8 / 50.5 |
| LAPS2 | 1.72 | 1.04 | 2 | 0 | 3 | 1 | 3 | -0.38 | -1.04 | 0.57 | 0.50 | 0.50 | 18.1 / 26.7 |
| LAPS3 | 2.46 | 0.74 | 3 | 0 | 3 | 2 | 3 | -1.25 | 0.87 | 0.82 | 0.52 | 0.26 | 1.8 / 60.1 |
| LAPS4 | 2.45 | 0.73 | 3 | 0 | 3 | 2 | 3 | -1.27 | 1.19 | 0.81 | 0.71 | 0.37 | 2.1 / 58.0 |
| LAPS5 | 2.39 | 0.76 | 3 | 0 | 3 | 2 | 3 | -1.27 | 1.39 | 0.79 | 0.55 | 0.25 | 3.6 / 53.0 |
| LAPS6 | 2.34 | 0.86 | 3 | 0 | 3 | 2 | 3 | -1.11 | 0.25 | 0.78 | 0.60 | 0.39 | 4.3 / 55.9 |
| LAPS7 | 2.61 | 0.65 | 3 | 0 | 3 | 2 | 3 | -1.81 | 3.28 | 0.87 | 0.49 | 0.19 | 1.8 / 69.0 |
| LAPS8 | 2.23 | 0.93 | 3 | 0 | 3 | 2 | 3 | -0.94 | -0.23 | 0.74 | 0.42 | 0.24 | 6.4 / 52.0 |
| LAPS9 | 2.32 | 0.87 | 3 | 0 | 3 | 2 | 3 | -1.17 | 0.52 | 0.77 | 0.44 | 0.32 | 5.7 / 53.7 |
| LAPS10 | 2.43 | 0.73 | 3 | 0 | 3 | 2 | 3 | -1.31 | 1.57 | 0.81 | 0.58 | 0.29 | 2.8 / 55.5 |
| LAPS11 | 2.55 | 0.65 | 3 | 0 | 3 | 2 | 3 | -1.45 | 2.02 | 0.85 | 0.49 | 0.16 | 1.4 / 63.0 |
| LAPS12 | 2.22 | 0.82 | 2 | 0 | 3 | 2 | 3 | -0.87 | 0.14 | 0.74 | 0.61 | 0.38 | 4.3 / 43.4 |
| LAPS13 | 2.47 | 0.73 | 3 | 0 | 3 | 2 | 3 | -1.32 | 1.27 | 0.82 | 0.60 | 0.28 | 2.1 / 59.4 |
| LAPS14 | 2.89 | 0.39 | 3 | 0 | 3 | 3 | 3 | -4.52 | 24.20 | 0.96 | 0.61 | 0.09 | 0.1 / 91.5 |
| LAPS15 | 2.76 | 0.50 | 3 | 0 | 3 | 3 | 3 | -2.41 | 6.72 | 0.92 | 0.70 | 0.19 | 0.07 / 79.7 |
| LAPS16 | 2.83 | 0.45 | 3 | 0 | 3 | 3 | 3 | -3.28 | 12.56 | 0.94 | 0.69 | 0.13 | 0.1 / 86.5 |
| LAPS17 | 2.60 | 0.63 | 3 | 0 | 3 | 2 | 3 | -1.57 | 2.34 | 0.86 | 0.54 | 0.18 | 1.1 / 66.9 |
| LAPS18 | 2.84 | 0.43 | 3 | 0 | 3 | 3 | 3 | -3.35 | 13.93 | 0.94 | 0.69 | 0.13 | 0.1 / 86.5 |
| LAPS19 | 2.84 | 0.41 | 3 | 0 | 3 | 3 | 3 | -3.35 | 14.91 | 0.94 | 0.70 | 0.13 | 0.1 / 86.1 |
| LAPS20 | 2.94 | 0.31 | 3 | 0 | 3 | 3 | 3 | -7.17 | 56.91 | 0.98 | 0.64 | 0.05 | 0.1 / 96.4 |
| LAPS21 | 2.86 | 0.54 | 3 | 0 | 3 | 3 | 3 | -4.47 | 19.51 | 0.95 | 0.43 | 0.08 | 2.8 / 93.2 |
| LAPS22 | 2.88 | 0.37 | 3 | 0 | 3 | 3 | 3 | -4.38 | 24.63 | 0.96 | 0.70 | 0.09 | 0.1 / 90.4 |
| LAPS23 | 2.82 | 0.45 | 3 | 0 | 3 | 3 | 3 | -3.04 | 11.17 | 0.94 | 0.71 | 0.16 | 0.1 / 84.7 |
| *Russia (N=236)* | | | | | | | | | | | | | |
| LAPS1 | 1.88 | 1.02 | 2 | 0 | 3 | 1 | 3 | -0.48 | -0.93 | 0.62 | 0.50 | 0.65 | 12.6 / 34.3 |
| LAPS2 | 2.27 | 0.79 | 2 | 0 | 3 | 2 | 3 | -0.93 | 0.34 | 0.75 | 0.31 | 0.50 | 3.4 / 45.6 |
| LAPS3 | 1.90 | 0.92 | 2 | 0 | 3 | 1 | 3 | -0.31 | -0.92 | 0.63 | 0.34 | 0.49 | 6.5 / 31.7 |
| LAPS4 | 1.83 | 1.06 | 2 | 0 | 3 | 1 | 3 | -0.39 | -1.13 | 0.61 | 0.59 | 0.73 | 14.8 / 35.2 |
| LAPS5 | 1.75 | 0.97 | 2 | 0 | 3 | 1 | 3 | -0.17 | -1.04 | 0.58 | 0.32 | 0.46 | 10.4 /27.4 |
| LAPS6 | 1.56 | 1.06 | 2 | 0 | 3 | 1 | 2 | -0.02 | -1.25 | 0.52 | 0.52 | 0.64 | 19.6 / 24.8 |
| LAPS7 | 2.12 | 0.96 | 2 | 0 | 3 | 1 | 3 | -0.66 | -0.79 | 0.70 | 0.26 | 0.40 | 6.1 / 47.0 |
| LAPS8 | 1.91 | 1.00 | 2 | 0 | 3 | 1 | 3 | -0.47 | -0.92 | 0.63 | 0.45 | 0.61 | 10.9 / 35.2 |
| LAPS9 | 1.44 | 1.11 | 1 | 0 | 3 | 0 | 2 | 0.05 | -1.35 | 0.48 | 0.36 | 0.46 | 26.5 / 22.6 |
| LAPS10 | 1.79 | 0.98 | 2 | 0 | 3 | 1 | 3 | -0.31 | -0.97 | 0.59 | 0.44 | 0.61 | 11.7 / 28.7 |
| LAPS11 | 1.77 | 1.01 | 2 | 0 | 3 | 1 | 3 | -0.22 | -1.14 | 0.59 | 0.38 | 0.52 | 12.2 / 30.9 |
| LAPS12 | 1.89 | 0.93 | 2 | 0 | 3 | 1 | 3 | -0.48 | -0.66 | 0.63 | 0.46 | 0.64 | 9.6 / 29.6 |
| LAPS13 | 1.85 | 1.07 | 2 | 0 | 3 | 1 | 3 | -0.46 | -1.06 | 0.61 | 0.50 | 0.63 | 15.7 / 35.2 |
| LAPS14 | 2.49 | 0.74 | 3 | 0 | 3 | 2 | 3 | -1.39 | 1.30 | 0.83 | 0.26 | 0.53 | 2.3 / 62.6 |
| LAPS15 | 2.22 | 0.93 | 3 | 0 | 3 | 2 | 3 | -0.94 | -0.17 | 0.74 | 0.47 | 0.70 | 6.5 / 50.4 |
| LAPS16 | 2.32 | 0.79 | 2 | 0 | 3 | 2 | 3 | -1.10 | 0.80 | 0.77 | 0.36 | 0.65 | 3.9 / 48.7 |
| LAPS17 | 2.22 | 0.81 | 2 | 0 | 3 | 2 | 3 | -0.71 | -0.34 | 0.74 | 0.25 | 0.47 | 2.6 / 43.9 |
| LAPS18 | 2.16 | 0.87 | 2 | 0 | 3 | 2 | 3 | -0.75 | -0.28 | 0.72 | 0.45 | 0.72 | 4.8 / 42.6 |
| LAPS19 | 2.53 | 0.72 | 3 | 0 | 3 | 2 | 3 | -1.52 | 1.80 | 0.84 | 0.31 | 0.64 | 2.2 / 64.8 |
| LAPS20 | 2.62 | 0.65 | 3 | 0 | 3 | 2 | 3 | -1.68 | 2.18 | 0.87 | 0.24 | 0.56 | 0.1 / 71.3 |
| LAPS21 | 2.20 | 1.03 | 3 | 0 | 3 | 2 | 3 | -1.04 | -0.25 | 0.73 | 0.41 | 0.52 | 11.7 / 54.3 |
| LAPS22 | 2.48 | 0.75 | 3 | 0 | 3 | 2 | 3 | -1.29 | 0.82 | 0.82 | 0.32 | 0.64 | 1.7 / 62.2 |
| LAPS23 | 2.31 | 0.84 | 3 | 0 | 3 | 2 | 3 | -1.03 | 0.20 | 0.77 | 0.45 | 0.75 | 3.9 / 52.6 |

*Notes*. Sk – Skew. Ku – Kurtosis. Diff – difficulty. i.e. difference of average score divided by range (maximal possible score maxscore minus minimal possible score minscore). Disc – discrimination (difference between the percent correct in the upper and lower third of the respondents tested). ITC – item-total correlation (correlation between item score and overall test score). L/H scores, % – percentage frequency of lowest or highest possible score achieved by respondents.
