# Supplementary material for: The Brief Lexington Attachment to Pets Scale: measurement invariance in India, Italy, Poland, and Russia
Source: BMC Psychol. 2025 Jul 9;13:754. doi: 10.1186/s40359-025-03080-6 (PMC12239344; doi:10.1186/s40359-025-03080-6)
Supplement: Supplementary file 2 — Additional file 2. Goodness-of-fit indices for the original and modified LAPS models in different cultures. The Table [file 40359_2025_3080_MOESM2_ESM.doc]

**Additional file 2.**

**Goodness-of-fit indices for the original and modified LAPS models in different cultures**

| Model | *χ^2^*/*df* | CFI | TLI | RMSEA (90% CI) | SRMR | Range of factor loadings, abs |
| --- | --- | --- | --- | --- | --- | --- |
| *Model 1:* Original 3-factor correlated model | | | | | | |
| India (*N* = 138) | 359.37/227*** | 0.880 | 0.866 | 0.065  (0.053; 0.077) | 0.066 | |0.26-0.83| |
| Italy (*N* = 153) | 402.10/227*** | 0.877 | 0.863 | 0.071  (0.060; 0.082) | 0.066 | |0.38-0.81| |
| Poland (*N* = 306) | 801.09/227*** | 0.819 | 0.799 | 0.091  (0.085; 0.097) | 0.086 | |0.43-0.90| |
| Russia (*N* = 236) | 556.88/227*** | 0.813 | 0.792 | 0.078  (0.071; 0.086) | 0.070 | |0.33-0.78| |
| *Model 2:* 3-factor correlated model with items 3, 6, 7, 12, and 22 removed | | | | | | |
| India (*N* = 138) | 150.93/132 | 0.973 | 0.969 | 0.032  (0.000; 0.052) | 0.059 | |0.27-0.84| |
| Italy (*N* = 153) | 172.20/132* | 0.959 | 0.952 | 0.045 (0.024; 0.062) | 0.050 | |0.37-0.81| |
| Poland (*N* = 306) | 342.48/132*** | 0.906 | 0.891 | 0.072 (0.064; 0.080) | 0.068 | |0.42-0.93| |
| Russia (*N* = 236) | 249.60/132*** | 0.901 | 0.885 | 0.062 (0.051; 0.072) | 0.058 | |0.29-0.75| |
| Models | χ2/df | CFI | TLI | RMSEA (90% CI) | SRMR | Range of factor loadings, abs |
| *Model 3:* Unidimensional model (23 items) | | | | | | |
| India (*N* = 138) | 382.05/230*** | 0.862 | 0.848 | 0.069  (0.058; 0.081) | 0.067 | |0.26-0.81| |
| Italy (*N* = 153) | 447.71/230*** | 0.847 | 0.831 | 0.079  (0.068; 0.089) | 0.068 | |0.35-0.80| |
| Poland (*N* = 153) ^a^ | 987.37/230*** | 0.762 | 0.738 | 0.104  (0.098; 0.110) | 0.082 | |0.32-0.84| |
| Russia (*N* = 118) ^a^ | 603.59/230*** | 0.788 | 0.767 | 0.083  (0.076; 0.090) | 0.071 | |0.35-0.76| |
| *Model 4:* Final unidimensional model (11 items) | | | | | | |
| India (*N* = 138) | 39.80/44 | 1.000 | 1.000 | 0.000  (0.000; 0.043) | 0.045 | |0.29-0.84| |
| Italy (*N* = 153) | 52.90/44 | 0.984 | 0.980 | 0.036 (0.000; 0.067) | 0.044 | |0.48-0.81| |
| Poland (*N* = 153) ^a^ | 62.83/44* | 0.974 | 0.967 | 0.053 (0.022; 0.078) | 0.047 | |0.31-0.81| |
| Russia (*N* = 118) ^a^ | 55.27/44 | 0.964 | 0.955 | 0.047 (0.000; 0.079) | 0.056 | |0.41-0.89| |

*Note*. χ^2^ = chi-square statistic; df = degrees of freedom; CFI = comparative fit index; TLI = Tucker–Lewis index; RMSEA = root mean square error of approximation; SRMR = standardized root mean square residual. *** = χ^2^ p-value < 0.001. ^a^ = In models 3 and 4, the entire Polish and Russian samples were divided in half for conducting separate EFAs and CFAs (see details in the Searching for a New Optimal Structure for LAPS section). Absolute values of the range of factor loadings are given, since items 8 and 21 load negatively on their scale.
