# Supplementary material for: The Brief Lexington Attachment to Pets Scale: measurement invariance in India, Italy, Poland, and Russia
Source: BMC Psychol. 2025 Jul 9;13:754. doi: 10.1186/s40359-025-03080-6 (PMC12239344; doi:10.1186/s40359-025-03080-6)
Supplement: Supplementary file 3 — Additional file 3. Factor loadings of the Lexington Attachment to Pets Scale items in different countries for a two-factor model. The Table [file 40359_2025_3080_MOESM3_ESM.docx]

**Additional file 3.**

**Factor loadings of the Lexington Attachment to Pets Scale items in different countries for a two-factor model**

|  | India | | Italy | | Poland | | Russia | |
| --- | --- | --- | --- | --- | --- | --- | --- | --- |
| Item | F1 | F2 | F1 | F2 | F2 | F1 | F1 | F2 |
| LAPS1 | 0.60 |  | 0.38 | 0.45 | 0.13 | 0.59 | 0.61 | 0.16 |
| LAPS2 | 0.44 | 0.12 | 0.28 | 0.44 | -0.21 | 0.70 | 0.33 | 0.49 |
| LAPS3 | 0.61 |  | 0.20 | 0.46 |  | 0.64 | 0.30 | 0.39 |
| LAPS4 | 0.64 |  |  | 0.79 | 0.10 | 0.76 | 0.69 |  |
| LAPS5 | 0.19 | 0.48 | 0.27 | 0.25 | 0.18 | 0.58 | 0.28 |  |
| LAPS6 | 0.31 | 0.41 | -0.15 | 0.85 |  | 0.73 | 0.67 | -0.14 |
| LAPS7 | 0.32 | -0.24 | 0.55 | -0.12 | 0.35 | -0.21 | 0.45 | -0.24 |
| LAPS8 | -0.24 |  | -0.30 | -0.31 | -0.27 | -0.32 | -0.59 | -0.12 |
| LAPS9 |  | 0.44 |  | 0.47 | 0.13 | 0.53 | 0.53 | -0.16 |
| LAPS10 | 0.46 | 0.28 | 0.36 | 0.31 | 0.26 | 0.36 | 0.66 |  |
| LAPS11 | 0.13 | 0.51 | 0.63 | -0.11 | 0.25 | 0.17 | 0.57 | -0.39 |
| LAPS12 | 0.35 | 0.32 | 0.28 | 0.47 | -0.12 | 0.79 | 0.65 | 0.12 |
| LAPS13 | 0.49 | 0.17 | 0.56 | 0.19 | 0.12 | 0.60 | 0.65 | -0.19 |
| LAPS14 | 0.80 | -0.11 | 0.24 | 0.40 | 0.44 | 0.20 | 0.30 | 0.40 |
| LAPS15 | 0.66 |  | 0.47 | 0.41 | 0.62 | 0.26 | 0.65 | 0.14 |
| LAPS16 | 0.69 |  | 0.65 | 0.20 | 0.77 |  | 0.53 | 0.12 |
| LAPS17 | 0.59 | -0.24 | 0.45 | 0.13 | 0.61 |  | 0.48 | -0.14 |
| LAPS18 | 0.79 |  | 0.56 | 0.24 | 0.84 |  | 0.75 |  |
| LAPS19 | 0.75 |  | 0.80 |  | 0.64 | 0.20 | 0.55 | 0.16 |
| LAPS20 | 0.80 |  | 0.50 | 0.12 | 0.89 |  | 0.38 | 0.40 |
| LAPS21 | -0.49 | 0.27 | -0.57 |  | -0.89 |  | -0.48 |  |
| LAPS22 | 0.79 |  | 0.78 |  | 0.85 |  | 0.59 | 0.10 |
| LAPS23 | 0.61 |  | 0.27 | 0.53 | 0.56 | 0.35 | 0.78 |  |
| Сumulative Explained Variances | **0.31** | **0.37** | **0.21** | **0.36** | **0.25** | **0.45** | **0.31** | **0.36** |

*Notes*. F1 — first factor, F2 — second factor.
