# Supplementary material for: The Brief Lexington Attachment to Pets Scale: measurement invariance in India, Italy, Poland, and Russia
Source: BMC Psychol. 2025 Jul 9;13:754. doi: 10.1186/s40359-025-03080-6 (PMC12239344; doi:10.1186/s40359-025-03080-6)
Supplement: Supplementary file 4 — Additional file 4. The Brief Lexington Attachment to Pets Scale: Extended English, Corresponding Reduced English, Italian, Polish, and Russian Versions. The Table [file 40359_2025_3080_MOESM4_ESM.docx]

**Additional file 4.**

**The Brief Lexington Attachment to Pets Scale: Extended English, Corresponding Reduced English, Italian, Polish, and Russian Versions**

**Instruction:** The questionnaire measures the attitude of people to their pet. Please rate your level of agreement with each statement by placing a ✔ in the box with the most appropriate option. Please answer each statement based on your own feelings and experience.

**Istruzione:** Pensando al tuo animale domestico, indica quanto sei in accordo o in disaccordo con le seguenti affermazioni.

**Instrukcja:** Proszę określ, czy zgadzasz się, czy nie zgadzasz z kilkoma krótkimi stwierdzeniami na temat Twojego ulubionego zwierzaka domowego. Przy każdym stwierdzeniu proszę zaznaczyć, czy się z nim zdecydowanie zgadzasz, trochę się zgadzasz, trochę się nie zgadzasz, czy zdecydowanie się nie zgadzasz.

**Инструкция:** Опросник измеряет отношение людей к своему питомцу. Пожалуйста, оцените степень вашего согласия с каждым утверждением, поставив ✔ в поле с наиболее подходящим вариантом. Пожалуйста, ответьте на каждое утверждение, основываясь на собственных ощущениях и опыте.

|  |  | **The Brief Lexington Attachment to Pets Scale** | | | |
| --- | --- | --- | --- | --- | --- |
| **Item code** | **The Lexington Attachment to Pets Scale**  **English wording** | **English wording** | **Italian wording** | **Polish wording** | **Russian wording** |
| 1 | My pet means more to me than any of my friends | My pet means more to me than any of my friends | Il mio cane significa molto per me, più di qualsiasi amico/a | Mój zwierzak znaczy dla mnie więcej niż którykolwiek z moich przyjaciół | Мой питомец значит для меня больше, чем некоторые из моих друзей |
| 2 | Quite often I confide in my pet | – | – | – | – |
| 3 | I believe that pets should have the same rights and privileges as family members | – | – | – | – |
| 4 | I believe my pet is my best friend | – | – | – | – |
| 5 | Quite often, my feelings toward people are affected by the way they react to my pet | – | – | – | – |
| 6 | I love my pet because he/she is more loyal to me than most of the people in my life | – | – | – | – |
| 7 | I enjoy showing other people pictures of my pet | – | – | – | – |
| 8 rev | I think my pet is just a pet | I think my pet is just a pet | Penso che il mio animale domestico sia solo un animale | Uważam, że mój zwierzak to po prostu zwierzę | Я считаю, что мой питомец – это просто домашнее животное |
| 9 | I love my pet because it never judges me | – | – | – | – |
| 10 | My pet knows when I'm feeling bad | My pet knows when I'm feeling bad | Il mio animale domestico sa quando sto male | Mój zwierzak wie, kiedy czuję się źle | Мой питомец знает, когда мне плохо |
| 11 | I often talk to other people about my pet | I often talk to other people about my pet | Spesso parlo agli altri del mio animale domestico | Często rozmawiam z innymi o moim zwierzaku | Я часто говорю с другими людьми о своем питомце |
| 12 | My pet understands me | – | – | – | – |
| 13 | I believe that loving my pet helps me stay healthy | I believe that loving my pet helps me stay healthy | Credo che amare il mio animale domestico mi aiuti a rimanere in salute | Wierzę, że miłość do mojego zwierzaka pomaga mi zachować zdrowie | Я верю, что любовь к моему питомцу помогает мне оставаться здоровым |
| 14 | Pets deserve as much respect as humans do | Pets deserve as much respect as humans do | Gli animali domestici meritano rispetto tanto quanto gli esseri umani | Zwierzaki zasługują na tyle samo szacunku, co ludzie | Домашние животные заслуживают того же уважения, что и люди |
| 15 | My pet and I have a very close relationship | My pet and I have a very close relationship | Io e il mio animale domestico abbiamo un rapporto molto stretto | Mój zwierzak i ja mamy bardzo bliską relację | Мы очень близки с моим питомцем |
| 16 | I would do almost anything to take care of my pet | – | – | – | – |
| 17 | I play with my pet quite often | I play with my pet quite often | Gioco abbastanza spesso con il mio animale domestico | Dość często bawię się z moim zwierzakiem | Я часто играю со своим питомцем |
| 18 | I consider my pet to be a great companion | I consider my pet to be a great companion | Considero il mio animale domestico un ottimo compagno | Uważam mojego zwierzaka za wspaniałego towarzysza | Я считаю своего питомца отличным компаньоном |
| 19 | My pet makes me feel happy | My pet makes me feel happy | Il mio animale domestico mi rende felice | Mój zwierzak sprawia, że jestem szczęśliwy/a | Мой питомец делает меня счастливым(ой) |
| 20 | I feel that my pet is a part of my family | – | – | – | – |
| 21 | I am not very attached to my pet | – | – | – | – |
| 22 | Owning a pet adds to my happiness | – | – | – | – |
| 23 | I consider my pet to be a friend | I consider my pet to be a friend | Considero il mio animale domestico un amico | Uważam mojego zwierzaka za przyjaciela | Я считаю питомца своим другом |

*Note.* Responses were made on a four-point scale: from 0 (Strongly disagree; Completamente in disaccordo; Zdecydowanie się nie zgadzam; Совершенно не согласен(а) to 3 (Strongly agree; Completamente d'accordo; Zdecydowanie się zgadzam; Полностью согласен(на)). Rev – reversed item. To get the overall score on the scale, add up the scores of each item.
